# Association between pre-chemotherapy serum levels of vitamin D and clinicopathologic findings in gastric cancer

**DOI:** 10.22088/cjim.11.3.290

**Published:** 2020-05

**Authors:** Akbar Hedayatizadeh-Omran, Ghasem Janbabaei, Reza Alizadeh-navaei, Omolbanin Amjadi, Jeyran Mahdavi Izadi, Versa Omrani-Nava

**Affiliations:** 1 Gastrointestinal Cancer Research Center, Mazandaran University of Medical Sciences, Sari, Iran

**Keywords:** Gastric cancer, Vitamin D, ELISA

## Abstract

**Background:**

To examine the serum levels of vitamin D in newly diagnosed gastric cancer (GC) patients compared with normal subjects and any possible association with prognostic variables.

**Methods:**

One-hundred subjects (50 GC and 50 controls) were enrolled and serum vitamin D levels were assessed using ELISA. Based on two definitions, vitamin D was classified as a sufficient level (≥30 ng/dL) and optimal level (25-80 ng/dL). The χ^2^and unpaired t-test was used for data analysis with a significance level of 0.05.

**Results:**

The mean serum levels of vitamin D in patients and controls were 26.86 (±14.6) and 31.72 (±13.4), respectively (P=0.09). The prevalence of vitamin D insufficiency and deficiency was higher in GC cases than controls (P=0.045 if sufficient level ≥30 and P=0.065 if sufficient level ≥25). According to histological grade analysis, grade 3 patients (poorly differentiated) were found with significantly lower vitamin D concentrations in serum than grade 1 and 2 subjects (22.25 vs 33.29 ng/dL, P=0.021). No significant differences were observed between the two groups in pathological tumor-node-metastasis (pTNM) stages, distant metastasis, and location of the tumor.

**Conclusion:**

Higher prevalence of vitamin D insufficiency and deficiency in GC patients may reflect its role in malignancy; however, further studies are needed to confirm this relationship and any possible benefits to the patients.

Vitamin D, better to be classified as a hormone, is a lipid-soluble nutrient. The human body has access to it in two main ways; by producing from a cholesterol-derived substance (7‐dehydrocholesterol) to previtamin D_3_ under the sun's ultraviolet B (UVB) rays and by receiving from foods or supplements (1). Vitamin D deficiency is not specific to a particular geographic region and is a global issue. According to two recent meta-analyses, almost 50% of Iranians were deficient at vitamin D (2, 3). Different factors affect serum vitamin D levels, including gender, age, body mass index, dairy consumption, and weather/humidity conditions (4). Vitamin D mainly acts to maintain blood levels of calcium and phosphorus via increasing intestinal absorption and decreasing renal excretion, thus plays a major role in establishing normal growth and health of the musculoskeletal system. Beyond these classic functions, recent studies have shed light on functional characteristics of vitamin D in the immune system and inflammatory pathways which have led to enormous attention on the relationship between vitamin D and a wide range of diseases, such as autoimmune disorders, cardiovascular diseases, and cancers (5). Anti-neoplasm activities of vitamin D are guaranteed by vitamin D receptor (VDR), a transcription factor belonging to nuclear receptor superfamily. 

In-vitro studies have revealed that combination of vitamin D with chemotherapeutic agents promotes gastric cancer cell apoptosis via different mechanisms, including overexpression of apoptosis-associated proteins like Bax (BCL2 Associated X), P27 and P21 (6), PTEN (Phosphatase and tensin homolog) (7), and TN F-α (8). The interaction of VDR-vitamin D complex with IKK-β kinase (which is known to suppress NF-κB) leads to down-regulation of inflammatory cytokines like IL-8 and IL-6 (9). These inflammatory cytokines can aid development of tumors and also associated with prognosis (10). Hypovitaminosis D is associated with higher serum levels of inflammatory cytokines like interleukin 6, 8, and 18 (11). Also, supplementation with vitamin D modulates IL-1 and IL-6 concentrations (12). VDR also inhibits NF-κB transcription factor, a critical mediator of inflammatory responses. In Iran, gastric cancer is the first prevalent malignancy in men and the second in women. The geographical distribution of this cancer is not homogeneous in Iran and is high in the North and northwest regions (13). In addition to genetic predisposing factors, lifestyle, nutrition and microbial agents contribute to gastric cancer. Therefore, the hypothesis is that subjects with low or suboptimal levels of vitamin D are more likely to develop cancer. Given the discrepancies in existing surveys (14), we aimed to evaluate the serum levels of vitamin D in patients with gastric cancer compared to the non-cancerous control group and investigate any relationship with clinical data. 

## Methods

In this case-control study, newly diagnosed, treatment naïve gastric adenocarcinoma patients and cases with normal endoscopic features were enrolled after obtaining informed written consent (April-September 2018). Individuals who were on vitamin D supplementation in the previous six months and cases with renal or hepatic disorders were excluded. About 2 ml of venous blood was taken from individuals and transferred to serum-separating tubes. Samples were centrifuged at 2500 RPM for 15 min and serum aliquots were immediately stored at -20 for further analysis. Serum vitamin D was assessed by a commercial enzyme-linked immunosorbent assay kit (AccuBind, USA). Demographic and clinical data were also recorded for each participant. The research contents were approved by the Ethics Committee of Sari Imam Khomeini Hospital (IR.MAZUMS.IMAMHOSPITAL.REC.1397.3021). 

Statistical analysis: SPSS 18 and GraphPad Prism 8.0.1 were applied for data analysis. The χ^2^test was used to analyze qualitative data, and levels of vitamin D among different subgroups were evaluated by unpaired t-test. A p<0.05 was considered statistically significant.

## Results

Totally, one-hundred samples from fifty newly diagnosed GC cases and fifty cases with normal endoscopic features were enrolled. There were 35 (70%) males in GC group and 27 (54%) in control group (p=0.1). The mean age of patients and controls was 64.78±13.13 and 51.62±13.35, respectively.

**Figure 1:**
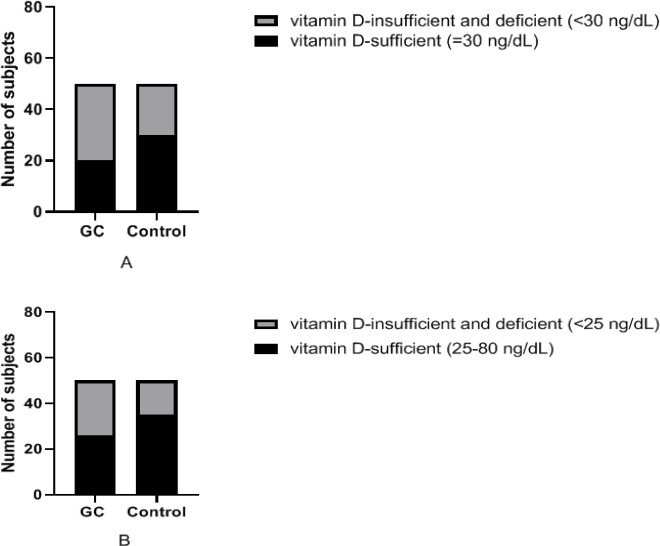
In section A, 40% of GC cases and 60% of controls had sufficient (≥30) vitamin D levels (P=0.045). Section B represents a similar analysis with a different classification level in which 52% of cases and 70% of controls were within sufficient levels of vitamin D (P=0.065).

 The mean serum levels of vitamin D in patients and controls were 26.86±14.6 and 31.72±13.4, respectively (P=0.09). Table 1 illustrates the classification of vitamin D status at three levels based on two different definitions (15). The prevalence of vitamin D insufficiency and deficiency was observed to be higher in GC cases than controls but this trend did not reach statistical significance at three-level comparisons (P=0.13 and 0.12). However, as shown in figure 1, a significant difference was observed between the two groups by dividing the results into two categories of sufficient and insufficient+deficient (P=0.045 and 0.065).

**Table 1:** Vitamin D levels in study groups

**P-value**	**Sufficient** **≥30**	**Insufficient** **21-29**	**Deficient** **<21**	**Categories ** **Groups**	
0.13	20(40%)	13 (26%)	17(34%)	GC cases	Definition 1
	30(60%)	8 (16%)	12 (24%)	controls	
P-value	Optimal 25-80	Mild-moderate deficiency 10-24	Severe deficiency 10>	Categories Groups	
0.12	26(52%)	18 (36%)	6 (12%)	GC cases	Definition 2
	35(70%)	13 (26%)	2 (4%)	controls	

Clinicopathologic data of patients are shown in table 2. Based on the location of the tumor, patients were divided into proximal (cardia, fundus, and body) or distal (antrum and pylorus) categories. Distal GC subjects had non-significant lower levels of serum vitamin D. In histological grade analysis, grade 3 patients (poorly differentiated) had significantly lower serum vitamin D concentrations (22.25 vs 33.29 ng/dL, P=0.021). No other associations were found regarding pTNM and distant metastasis and serum vitamin D.

**Table 2:** Association between clinicopathologic data and serum vitamin D concentration

**Variables (N, %)**	**Serum vitamin D ng/dL**	**P-value**
GC location		
Proximal (25, 50%) Distal (22, 50%)	29.74±13.46 24.28±16	0.22
Histological Grade		
well and moderately differentiated (17, 34%) Poorly differentiated (19, 38%)	33.29±13.5 22.25±13	0.021
pTNM		
I, II (11, 22%) III, IV (33, 66%)	28.47±15.2 27.90±15	0.9
Metastasis		
Positive (14, 28%) Negative (35, 70%)	25.07± 17.26 27.7±13.58	0.21

## Discussion

Heart diseases, cancers, and injuries are among the top leading causes of mortality in Iran (16, 17). Today, special attention is being paid to diet and nutrition as predisposing or preventative factors of cancer. In the meantime, vitamins are known to have antioxidative and anti-inflammatory properties and there is evidence of their role in preventing cancers (18). 

In the current study, we analyzed the serum concentration of vitamin D in 50 newly diagnosed chemotherapy-naïve GC patients and control subjects. Vitamin D insufficiency/ deficiency was found to be more prevalent in patients. Also, lower levels of vitamin D were associated with higher tumor grade. This is consistent with the results of Janbabai et al. (19) and Karthikayan et al. (20) in breast cancer patients. In contrast, Chao Ren et al. found no association between vitamin D status and tumor grade in Chinese GC cases (21). As mentioned in the introduction section, the biological functions of vitamin D is initiate by binding to VDR. VDR expression in malignant and non-malignant gastric tissues and its relationship between clinicopathological features was discussed by Yanghui Wen et al. VDR was significantly lower expressed in gastric cancer tissues compared to normal and pre-malignant ones. Poorly differentiated tissues exhibited lower expression than that in well and moderately differentiated samples (22). While evaluation of VDR was not included in our study, the conclusions are in line with the present study. A study on patients with pre-cancerous gastric lesions (gastric incomplete intestinal metaplasia) indicated a higher frequency of vitamin D insufficiency and deficiency among intestinal metaplasia cases. Also, serum levels of vitamin D were significantly lower in patients (19.7 ng/dL) compared to those in individuals without intestinal metaplasia (34.7 ng/dL) (23). 

MS El Shahawy et al. observed that the sufficient serum levels of vitamin D contribute to better eradication of *Helicobacter* pylori infection therefore, cases with chronic gastritis and vitamin D insufficiency showed higher rate of failure in eradication therapy (24). Given the role of this infection in developing gastric inflammation, ulcers and malignancy, mentioned findings may indicate the importance of adequate levels of vitamin D in preventing GC through elimination of infection (25). However studies that conducted on this issue and evaluated food and supplement intake by questionnaire as Mayne et al, reported no protective role for vitamin D on gastric cancer while other nutrients as vitamin E, C, B6, and beta-carotene showed an inverse association with gastric cancer risk (26). A randomized clinical trial revealed that the administration of daily vitamin D in patients with gastrointestinal epithelial carcinoma can lead to significantly longer 5-year relapse-free survival so this might highlight the anti-cancer properties of it (27) although, evaluating patients' survival has been beyond the aim of our study. In contrast, one study in Chinese population reported higher serum levels of vitamin D in patients with gastric cardia/non-cardia adenocarcinoma and oesophageal squamous cell carcinomas (ESCC) which was significantly higher in ESCC cases (28). One of the strengths of the current study is that sampling was done before treatment initiation as there is evidence on the effect of chemotherapy drugs on bone density and vitamin D levels (29). On the other hand and as limitations, the measurement was performed after the diagnosis of cancer and there was no information on vitamin D concentrations at pre-malignant and during tumor initiation stages. Also, cancer is also a multifactorial disease and a number of factors are among the predisposing agents, one of them might be vitamin D deficiency. 

In conclusion, we observed that vitamin D insufficiency/ deficiency was more prevalent in newly diagnosed GC patients compared to controls and patients with higher tumor grade had lower levels of it. Because the region studied (North of Iran) has a high risk of digestive system cancers (30), longitudinal studies to measure the association between levels of vitamin D and malignancy, as well as prognosis, can be of great benefit in clarifying this relationship.
